# Comparative Evaluation of Multiplex Real-Time PCR, Standard Urine Culture, and Rapid Nephelometric Screening in Patients with Complicated Urinary Tract Infections

**DOI:** 10.3390/diagnostics16060919

**Published:** 2026-03-19

**Authors:** Milena Yancheva Rupcheva, Kostadin Kostadinov, Radoslav Tashev, Petya Markova, Violeta Zheleva, Maritza Chterev, Mariya Atanasova, Michael M. Petrov, Marianna Murdjeva

**Affiliations:** 1 Department of Medical Microbiology and Immunology “Prof. Dr. Elissay Yanev”, Faculty of Medicine, Medical University of Plovdiv, 4002 Plovdiv, Bulgaria; radoslav.tashev@mu-plovdiv.bg (R.T.); mariya.atanasova@mu-plovdiv.bg (M.A.); michael.petrov@mu-plovdiv.bg (M.M.P.); 2 Research Institute of Medical University of Plovdiv (RIMU), 4002 Plovdiv, Bulgaria; kostadinr.kostadinov@mu-plovdiv.bg; 3 Laboratory of Microbiology, University Hospital St. George, 4002 Plovdiv, Bulgaria; 4 Department of Social Medicine and Public Health, Faculty of Public Health, Medical University of Plovdiv, 4002 Plovdiv, Bulgaria; 5 Department of Paediatrics, Medical University of Plovdiv, 4002 Plovdiv, Bulgaria; petya.markova@mu-plovdiv.bg; 6 Second Department of Internal Medicine, Nephrology Section, Medical University of Plovdiv, 4002 Plovdiv, Bulgaria; violeta.zheleva@mu-plovdiv.bg; 7 Faculty of Medicine, Medical University of Plovdiv, 4002 Plovdiv, Bulgaria; maritzachterev@gmail.com; 8 Laboratory of Virology, University Hospital St. George, 4000 Plovdiv, Bulgaria; 9 Department of Microbiology and Virology, Faculty of Pharmacy, Medical University of Pleven, 5800 Pleven, Bulgaria; mmurdjeva@yahoo.com; 10 Institute for Innovation and Smart Technology (IIST), University of Telecommunication and Posts, 1700 Sofia, Bulgaria

**Keywords:** method agreement, diagnostics, multiplex PCR, pyelonephritis, standard urine culture, urinary tract infection

## Abstract

**Background/Objectives:** Microbiological confirmation of suspected complicated urinary tract infections (cUTIs) is challenging, particularly in patients previously exposed to antibiotics or when fastidious organisms are involved. Molecular assays detect microbial nucleic acids independently of bacterial viability and may therefore yield results that differ from conventional culture. This study compared microorganism detection patterns and inter-method agreement between multiplex real-time PCR (mPCR), standard urine culture SUC, and rapid nephelometric screening (Uroquattro HB&L). **Methods:** In a prospective single-centre study, urine samples from 72 hospitalized patients with clinical suspicion of cUTIs were analyzed using SUC, mPCR (Novaplex™ UTI panel), and the Uroquattro system. Detection rates were calculated for each method. Agreement between paired methods was evaluated using Cohen’s kappa, and paired differences in detection were assessed using McNemar’s and Cochran’s Q tests. **Results:** mPCR detected microorganisms in 83.3% of samples, compared with 47.2% for SUC and 42.6% for Uroquattro. Agreement between mPCR and SUC was fair (κ = 0.26), whereas SUC and Uroquattro demonstrated high concordance. mPCR identified a broader spectrum of organisms, including fastidious and polymicrobial findings that were not recovered by culture. Correlation between PCR cycle threshold values and colony counts was weak and not statistically significant. **Conclusions:** mPCR demonstrated a substantially higher microorganism detection frequency than culture-based methods; however, the assays target different biological characteristics, highlight bacterial nucleic acid versus viable growth, and should be interpreted as complementary rather than interchangeable. Conventional culture remains necessary for antimicrobial susceptibility testing and clinical decision-making. Further studies incorporating clinical outcome-based reference standards are required to determine the clinical relevance of molecular detection in cUTIs.

## 1. Introduction

Urinary tract infections are the most common bacterial infections acquired in both the community and hospitals. They account for millions of visits to the doctor each year and result in hospitalizations, placing them at a significant health and economic cost [1]. While common, their clinical management is guided by the distinction between uncomplicated and complicated UTIs (cUTIs). According to the European Association of Urology (EAU) and the Infectious Diseases Society of America (IDSA), cUTIs are defined by the presence of factors that increase the risk of treatment failure or serious outcomes, such as structural abnormalities of the urinary tract, male sex, pregnancy, or underlying comorbidities like diabetes and immunodeficiency [2]. The incidence of UTIs in women is higher than in men [3]. Recent studies within the Bulgarian hospital setting, specifically at University Hospital “St. George” in Plovdiv, have highlighted significant trends in etiology and rising antibiotic resistance among uropathogens [4]. In the United States, approximately 40% of women experience a UTI, making these infections the most frequent among them [5].

The diagnosis of urinary tract infections is based not only on clinical data, but also on the results of clinical, laboratory and microbiological examinations of the patient [6]. The most used microbiological methods include standard urine culture, standard biochemical examination, as well as semi-automated and automated methods (MALDI-TOF and Vitek 2 Compact) [7]. Over time, SUC has become the gold standard for diagnosis, as it allows the identification of uropathogens, the quantification of bacterial load, and the determination of phenotypic antibiotic susceptibility [8]. The IDSA and EAU define significant bacteriuria based on specific thresholds, typically ≥10^3^ CFU/mL for acute cystitis in women and ≥10^5^ CFU/mL for cUTIs or asymptomatic bacteriuria [2].

The use of highly sensitive molecular methods overcomes the challenge of distinguishing between asymptomatic bacteriuria and colonization. Consequently, SUC as a “gold standard” is increasingly debated in modern microbiology. It has some limitations, such as the result usually takes 24–72 h to obtain [9]. Also, the SUC protocol may miss difficult-to-grow or fastidious microorganisms such as *Actinobaculum shaalii* [10]. It also often fails to detect pathogens in cases where patients have already received antibiotic treatment [11,12]. In addition, polymicrobial infections are often overlooked or dismissed as sample contamination [13]. Extended urine culture protocols (EQUC), using microaerophilic conditions and longer incubation (48 h), have demonstrated that up to 67% of uropathogens missed by SUC can be detected; however, due to delayed results, EQUC has not been adopted in routine microbiological practice [9].

Currently, there are many methods that may upgrade SUC, such as Matrix-Assisted Laser Desorption/Ionization Time-of-Flight (MALDI-TOF), multiplex quantitative real-time PCR, and whole-genome sequencing. MALDI-TOF requires cultured microorganisms, but in cases of antibiotic therapy, even a single dose, urine may remain culture-negative, making this method insufficient in such cases [14,15].

Whole-genome sequencing is a laborious and expensive method and is not applicable in routine practice because bioinformatic analysis requires 2–5 days. It is useful for tracking closely related strains and detecting resistance genes [16,17]. However, this information could be obtained more quickly (approximately 3 h) by quantitative PCR analysis [11]. Quantitative PCR could also be an alternative to culture because, in addition to its identification, it is possible to quantitatively assess uropathogens based on the reported Ct value dependence [18,19].

Moreover, PCR has been shown to exhibit higher analytical detection rate than SUC in detecting polymicrobial and fastidious organisms that are difficult or impossible to culture [11], although its clinical specificity regarding active infection versus colonization remains a subject of ongoing discussion [20]. By providing more comprehensive diagnostic information in a shorter timeframe, PCR can support more precise antimicrobial therapy and enhance antimicrobial stewardship practices [20,21,22].

PCR has been reported to identify polymicrobial infections in 42% of cases compared with only 2.1% detected by culture (*p* < 0.01) in patients with complicated urinary tract infections (cUTIs) [11]. A meta-analysis further demonstrated that PCR achieves a sensitivity of approximately 99% and a specificity of 94% in UTI diagnosis, even when compared with next-generation sequencing (NGS) as a reference method [23]. More recently, targeted next-generation sequencing (tNGS) has demonstrated comparable or superior pathogen identification to both metagenomic NGS and traditional culture in multicentre evaluations, with substantially shorter turnaround times and enhanced detection of polymicrobial infections and antibiotic resistance genes [24]. However, the transition from culture to molecular panels introduces the risk of overdiagnosis.

The challenge in diagnosing UTIs is in patients who have taken antibiotics at home or immediately before seeing a doctor. In these cases, SUC often gives false negative results, necessitating more sensitive molecular methods for precise identification of the causative agent.

The Uroquattro HB&L system (Alifax, Italy) employs laser nephelometry to detect changes in light scattering caused by the metabolic activity and early growth of microorganisms in liquid culture medium, thereby providing a rapid screening result (approximately 3 h) that, like SUC, is dependent on bacterial viability [25].

Comparative analysis of the results of SUC and PCR analysis of urine is often found in the literature, but there is a lack of data on the effectiveness of using rapid nephelometric screening for bacterial growth compared to molecular diagnostics. The aim of our study was to perform a comparative analysis of the SUC and PCR methods and to evaluate the role of the rapid nephelometric method applied in combination with multiplex quantitative real-time PCR.

At present, the quantitative correlation between the metabolic activity of microorganisms, as measured by laser nephelometry (Uroquattro), and the actual number of copies of the genetic material (replicons) detected by qPCR is not yet fully understood. The lack of data on such a relationship highlights the need for studies analyzing the relationship between bacterial viability and their molecular detection.

A central challenge in UTI diagnostics is that microbiological detection does not necessarily equate to clinical infection. Culture identifies viable bacteria, whereas PCR detects bacterial DNA that may originate from active infection, colonization, or non-viable organisms after antibiotic therapy. Consequently, discordance between molecular and culture methods is expected, and the clinical interpretation of such discordance remains unclear. The present study therefore aimed not to determine diagnostic accuracy, but to compare microorganism detection patterns and agreement between mPCR, standard culture, and rapid screening in patients with clinically suspected complicated UTI.

## 2. Materials and Methods

This prospective comparative diagnostic study evaluated agreement between three microbiological detection methods. The study did not employ an independent clinical reference standard; therefore, analyses focused on detection rates and inter-method agreement rather than diagnostic accuracy. All laboratory analyses were performed blinded to the results of the other methods. The study was conducted in accordance with the Declaration of Helsinki and approved by the Ethics Committee of the Medical University of Plovdiv (Protocol No. 3/21.03.2024 regarding University Research Project No. 3.4.3/15.01.2024; approval date: 27 March 2024).

### 2.1. Population, Eligibility, and Unit of Analysis

During the study period from January 2024 to March 2025, 72 patients from 6 months to 92 years were selected. Patients were included based on clinical data of UTI diagnosed by nephrologists. Only the first isolate per patient per calendar year was used in the statistical analysis. To reduce confounding from repeated sampling and maintain independence of observations, deduplication was applied for each patient.

Participants were excluded from the study based on the following criteria: 1. Lack of informed consent from the patients or their legal guardians; 2. Absence of clinical symptoms, e.g., dysuria, urgency; 3. Urine collected via indwelling catheters; 4. Patients with spinal cord injuries or neurogenic bladder dysfunction; 5. Immunosuppressed patients such as the presence of primary or secondary immunodeficiency, including HIV infection, active malignancy under chemotherapy, treatment with systemic corticosteroids or other immunosuppressive agents, and a history of solid organ transplantation; 6. Inadequate sample volume (less than 5 mL); 7. Macroscopic haematuria (visible presence of blood in the urine); 8. Urine collected in non-sterile containers; 9. Failure to adhere to protocols for proper collection, transport, and storage of the samples; 10. Samples yielding three or more distinct organisms without a dominant isolate (as defined in Section 2.3) and with no clinical evidence supporting polymicrobial infection were considered probable contamination and excluded. Clinical evidence supporting polymicrobial infection was defined as the presence of one or more of the following: (a) a clinical diagnosis of acute pyelonephritis or urosepsis with systemic inflammatory signs (fever ≥ 38 °C, elevated CRP, or leucocytosis); (b) structural urinary tract abnormalities or indwelling devices predisposing to polymicrobial colonization; or (c) recurrent or persistent complicated UTI with documented prior polymicrobial episodes. This determination was made by the treating clinician in conjunction with the microbiologist at the time of laboratory reporting. Samples with three or more organisms in which a dominant pathogen was identifiable, or in which the clinical presentation met the above criteria for polymicrobial infection, were retained and analyzed at the species level.

### 2.2. Sample Collection

Mid-morning urine samples were collected in sterile containers after thorough cleansing of the external genitalia. All samples were transported to the laboratory at ambient temperature and processed within a maximum of 2 h of collection, in accordance with institutional standard operating procedures, to minimize the impact of pre-analytical variability on culture yield and nucleic acid integrity [26].

All samples were tested for bacterial growth by standard culture, rapid 3-h nephelometric screening using the HB&L Uroquattro apparatus (Alifax, Polverara (PD), Italy), and multiplex PCR analysis (Novaplex UTI, Seegene, Seoul, Republic of Korea) in 3 panels for 30 of the most common uropathogens.

### 2.3. Standard Urine Culture

All urine samples were cultured using a calibrated 1.76 μL (0.001 mL) dipstick and a semi-quantitative streak method on 5% Sheep Blood Agar (Cat. No. M1301, HiMedia Laboratories, Thane West, India), Eosin-Methylene Blue (EMB) medium (Cat. No. M022, HiMedia Laboratories, India) and for yeast, HiCrome^®^ Candida Differential Agar Plate (Cat. No. MP1297A, HiMedia Laboratories, India). The plates were incubated aerobically at 35–37 °C for 18–24 h. Yeast cultures were incubated at 30–35 °C for 24–48 h.

Colonies were counted in the primary band area and converted to CFU/mL = number of colonies × (1/loop volume in mL), i.e., multiplied by 10^3^ for a 1.76 μL loop. Bacterial culture with ≥176 colonies of a single morphotype were interpreted as ≥10^5^ CFU/mL; Colony count of 18–176 of a single morphological type were interpreted as 10^4^ CFU/mL; and <18 colonies of a single morphological type were interpreted as <10^4^ CFU/mL. When ≥2 different morphotypes were present without a dominant organism and at low counts (<10^4^ ÷ 10^5^ CFU/mL), the results were interpreted as probable contamination, and re-sampling was recommended. If a dominant organism exceeded the respective threshold with a negligible secondary population, the dominant isolate was reported. Polymicrobial cultures were analyzed at the species level. For the purpose of this study, a dominant isolate was operationally defined as a single morphotype present at ≥10^5^ CFU/mL when all secondary morphotypes were present at ≤10^4^ CFU/mL, that is, a minimum one-log differential between the dominant and secondary populations. When two or more morphotypes were present at comparable concentrations (within one log_10_ CFU/mL of each other) and no single organism predominated, the culture was classified as mixed flora without a dominant isolate. Identification after isolation was performed using standard biochemical methods (Remel, BioMérieux, Marcy-l’Étoile, France) and automated methods such as Matrix-Assisted Laser Desorption/Ionization Time-of-Flight Mass Spectrometry (MALDI-TOF Vitek MS) (BioMérieux, France) and Vitek 2 compact (BioMérieux, France). Samples with ≥3 organisms in which no dominant organism was identifiable and no clinical correlate supported polymicrobial infection were recorded as contamination and excluded per the eligibility criteria. Remaining polymicrobial samples were retained and analyzed at the species level, as polymicrobial detection constituted an outcome of interest.

### 2.4. HB&L UROQUATTRO System (Alifax, Polverara (PD), Italy)

The samples were subjected to standard screening for bacterial growth using the HB&L UROQUATTRO system (Alifax, Polverara (PD), Italy). Urine samples were inoculated into a vial containing broth (URO-QUICK Screening Kit, Catalogue No. SI 190.900, Alifax, Polverara (PD), Italy), following the manufacturer’s instructions. The screening time was set for 3 h.

### 2.5. mPCR Analysis

Automated genomic DNA extraction was performed using the SEEPREP32 system (Seegene, Seoul, Republic of Korea) in combination with the STARMag 96 ProPrep C Tube Kit (Cat No. EX00017T, Seegene, Seoul, Republic of Korea) following the manufacturer’s instructions. The whole extraction procedure took about 30 min. Simultaneous detection and identification of uropathogens were performed using the Novaplex™ Urinary Tract Infection (UTI) Panel Assays (Seegene, Seoul, Republic of Korea; Cat. Nos. R-SD10228W, R-SD10240W, and R-SD10241W for Panels 1, 2, and 3, respectively), which consist of three distinct panels targeting a broad spectrum of Gram-negative bacteria, Gram-positive bacteria, and fungi.

Panel 1 targeted the most common major Gram-negative pathogens, including *Escherichia coli*, *Klebsiella pneumoniae*, *Proteus mirabilis*, *Pseudomonas aeruginosa*, and *Enterobacter* spp. Panel 2 covered Gram-positive cocci and fungal pathogens: *Enterococcus faecalis*, *Enterococcus faecium*, *Staphylococcus aureus*, *Staphylococcus saprophyticus*, *Staphylococcus epidermidis*, *Streptococcus agalactiae* (Group B Streptococcus), *Actinobacullum* (*Actinotignum*) *schaalii*, *Candida albicans*, and other *Candida* spp. Panel 3 targeted additional clinically relevant organisms, including *Acinetobacter baumannii*, *Aerococcus urinae*, *Citrobacter freundii*, *Citrobacter koseri*, *Corynebacterium urealyticum*, *Morganella morganii*, *Pantoea agglomerans*, *Providencia stuartii*, and *Streptococcus anginosus*. A complete list of all microorganisms targeted by each panel is provided in Table A3.

The procedure was prepared in accordance with the manufacturer’s manual. Amplification was performed on a CFX96 Real-Time PCR Detection System (Bio-Rad, Hercules, CA, USA) using the specific thermal cycling profile recommended by the manufacturer. The results were analyzed using the Seegene Viewer software (Ver. 3.30.000), which interprets the cycle threshold (Ct) values and melting curves generated by the proprietary MuDT™ technology to identify specific pathogens. The whole mPCR procedure occupied about 2 h.

### 2.6. Statistical Analysis

Descriptive statistics were used to summarize the study population and laboratory findings. Continuous variables (e.g., age and laboratory parameters) are presented as medians with interquartile ranges (IQR), and categorical variables (e.g., sex, diagnosis, and symptoms) as counts and percentages.

For each diagnostic method, SUC, multiplex real-time PCR (mPCR), and Uroquattro screening, the microorganism detection rate was calculated as the proportion of samples with a positive result among all analyzed samples. Because no independent clinical reference standard was available, measures of diagnostic accuracy (sensitivity, specificity, and predictive values) were not calculated. Accordingly, the analyses represent a comparative method-agreement study rather than an assessment of diagnostic accuracy.

Agreement between methods was evaluated using Cohen’s kappa (κ) for paired binary outcomes (positive/negative detection). Kappa values were interpreted according to Landis and Koch criteria: <0.20 poor, 0.21–0.40 fair, 0.41–0.60 moderate, 0.61–0.80 substantial, and >0.80 almost perfect agreement.

Paired differences in microorganism detection between methods were assessed using McNemar’s test. Overall differences across the three paired methods were evaluated using Cochran’s Q test. Associations between PCR cycle threshold (Ct) values and colony counts in matched detections were explored using Spearman’s rank correlation coefficient (ρ).

Comparisons of continuous laboratory parameters between groups defined by culture positivity were performed using the Wilcoxon rank-sum test. All statistical tests were two-sided, and a *p*-value < 0.05 was considered statistically significant. Statistical analyses were performed using R version 4.5.2 (R Foundation for Statistical Computing, Vienna, Austria). Cohen’s kappa coefficients were calculated using the irr package (version 0.84.1), Wilson score confidence intervals using the binom package (version 1.1-1.1), and data manipulation and visualization were performed using the tidyverse suite of packages.

No formal a priori power calculation was performed, as the study was designed as an exploratory method-agreement investigation rather than a confirmatory trial. The sample size of 72 patients was determined by pragmatic recruitment of consecutive eligible patients over the 15-month study period. For the primary analysis, pairwise comparison of detection rates using McNemar’s test, a sample of 72 provides approximately 80% power to detect a difference of 20 percentage points in paired proportions at a two-sided α of 0.05, which is consistent with the magnitude of difference observed between mPCR and SUC (83.3% vs. 47.2%). For Cohen’s kappa, simulation studies indicate that a minimum of 50–60 observations is generally sufficient to estimate kappa with acceptable precision when the prevalence of the outcome is moderate [27,28]. The achieved sample size is therefore considered adequate for the primary method-level comparisons, although organism-specific subgroup analyses should be interpreted as exploratory and hypothesis-generating given the sparse cell counts for individual pathogens.

## 3. Results

### 3.1. Patient Characteristics and Overall Detection Rates of All Methods

A total of 72 patients were analyzed, with characteristics summarized in Table 1. The cohort was categorized based on whether any microorganisms were identified using at least one of three diagnostic methods (PCR, standard culture) and was screened for bacterial growing by Uroquattro screening. Patients with no microbial detection (*n* = 11) formed the Negative Detection group, while those with at least one positive test (*n* = 61) formed the Positive Detection group.

The overall median age was 58 years (IQR: 40–71), with no significant age difference between the two groups (Negative: 58 [25–71]; Positive: 58 [42–70]; *p* > 0.9). Women made up 74% of the entire cohort (*n* = 53), with similar proportions in each group (Negative: 82%, *n* = 9; Positive: 72%, *n* = 44; *p* = 0.7). Diagnoses were predominantly acute pyelonephritis (83%, *n* = 60), followed by acute cystitis (17%, *n* = 12), with no significant distributional difference between groups (*p* = 0.4).

Symptom frequency varied, with dysuria (81%, *n* = 58) and urine discoloration (82%, *n* = 59) being the most common, while enuresis (1.4%, *n* = 1) and haematuria (5.6%, *n* = 4) were rare. No individual symptoms differed significantly between the groups (all *p* > 0.2), although there were non-significant trends suggesting increased urgency in the Negative Detection group (45% vs. 28%, *p* = 0.3) and greater urine cloudiness in the Positive Detection group (70% vs. 55%, *p* = 0.3).

Laboratory parameters such as median white blood cell count (WBC: 8.7 × 10^9^/L, IQR: 6.5–10.8), hemoglobin (HGB: 125 g/L, IQR: 112–137), and C-reactive protein (CRP: 8 mg/L, IQR: 5–31) showed no significant differences (all *p* ≥ 0.4). Similarly, urine specific gravity (1.015 g/L, IQR: 1.010–1.025) and pH (5.50, IQR: 5.00–6.00) did not differ significantly (*p* = 0.4 and 0.8, respectively).

**Figure 1:**
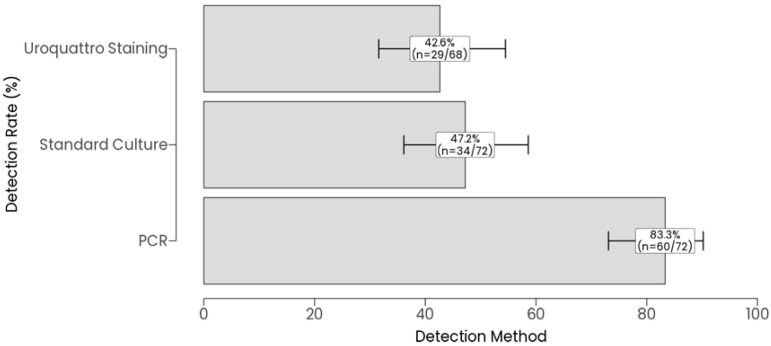
Microorganism Detection Rates by Method. Bar plot of detection rates for PCR, standard culture, and Uroquattro screening, with 95% confidence intervals (Wilson score method).

Detection rates of microorganisms by three methods: PCR, standard culture, and Uroquattro screening, were evaluated in a cohort of 72 patients, with Uroquattro screening performed on a subset of 68 samples for insufficient residual volume after culture and PCR aliquoting. As shown in Figure 1, PCR detected microorganisms in 83.3% of 72 samples (*n* = 60, 95% CI: 73.1–90.2%), detected a standard culture in 47.2% of 72 samples (*n* = 34, 95% CI: 36.1–58.6%), and performed Uroquattro screening in 42.6% of 68 samples (*n* = 29, 95% CI: 31.6–54.5%). These rates, with their 95% Wilson score confidence intervals, highlight PCR’s markedly higher detection rate compared to both standard culture and Uroquattro screening, which exhibited overlapping CIs. Pairwise McNemar’s tests, conducted on the 68 samples with complete data for all three methods, confirmed significant differences in detection frequency: mPCR yielded a higher positivity rate than standard culture (*p* < 0.01) and Uroquattro screening (*p* < 0.01), while the difference between standard culture and Uroquattro screening was not significant (*p* = 0.371). Cochran’s Q test further supported an overall significant difference across the methods (Q = 89.69, df = 2, *p* < 0.01).

Among the 60 mPCR-positive samples, 47 (78.3%) yielded ≥2 distinct microorganisms (polymicrobial detection), whereas only 4 of 72 samples (5.6%) were classified as polymicrobial by SUC (*p* < 0.01). The distribution of organism counts per mPCR-positive sample was as follows: one organism in 13 samples (21.7%), two in 17 (28.3%), three in 11 (18.3%), four in 10 (16.7%), and five or more in 9 (15.0%). Among the 47 polymicrobial mPCR samples, SUC was negative in 21 (44.7%), yielded a single isolate in 22 (46.8%), and detected ≥2 organisms in 4 (8.5%). Organism-level concordance was assessed for the 26 culture-positive samples within this group: partial concordance, defined as at least one shared species between mPCR and SUC, was observed in 24 cases (92.3%), while complete discordance was present in 2 cases (7.7%). The most frequently co-detected organisms in polymicrobial mPCR samples were *Escherichia coli* (present in 24/47, 51.1%), *Enterococcus faecalis* (23/47, 48.9%), and *Actinobaculum schaalii* (22/47, 46.8%), followed by *Streptococcus anginosus* (12/47, 25.5%) and *Aerococcus urinae* (9/47, 19.1%). The most recurrent combination was *E. coli* with *E. faecalis* (observed in 3 samples). Overall, 41 of the 47 polymicrobial mPCR detections (87.2%) occurred in patients with a clinical diagnosis of acute pyelonephritis. The cross-tabulation of mPCR detection status against SUC results is presented in Table A1.

Method discordance was stratified into four categories based on binary mPCR and SUC results across all 72 samples (Table A2). Concordant positive results (both methods positive) were observed in 33 samples (45.8%), concordant negative results (both methods negative) in 11 (15.3%), PCR-positive/culture-negative discordance (PCR+/SUC−) in 27 (37.5%), and the reverse pattern (PCR−/SUC+) in 1 (1.4%). The PCR+/SUC− pattern was thus the predominant discordance type. Patient characteristics were compared between the PCR+/SUC− and concordant-positive groups: median age was 56 years (IQR: 14–69) versus 61 years (IQR: 49–75) (*p* = 0.252), the proportion of females was 66.7% versus 75.8% (*p* = 0.567), and acute pyelonephritis was diagnosed in 85.2% versus 84.8% of patients (*p* = 1.000). Median CRP was 9.8 mg/L (IQR: 5.6–73.8) in the PCR+/SUC− group compared with 6.3 mg/L (IQR: 3.0–21.3) in the concordant-positive group (*p* = 0.097), and median WBC counts were 9.4 × 10^9^/L (IQR: 6.9–11.6) versus 8.1 × 10^9^/L (IQR: 6.4–9.4) (*p* = 0.119). None of these differences reached statistical significance. The most frequently detected organisms among PCR+/SUC− samples were *Actinobaculum schaalii* (12/27, 44.4%), *Escherichia coli* (10/27, 37.0%), *Enterococcus faecalis* (9/27, 33.3%), and *Streptococcus anginosus* (6/27, 22.2%). Uroquattro screening was positive in only 1 of 25 PCR+/SUC− samples with available data (4.0%), compared with 27 of 31 concordant-positive samples (87.1%). Reverse discordance (PCR−/SUC+) was observed in a single case involving *Enterococcus faecium* recovered by culture with a concurrent negative mPCR result, possibly reflecting a concentration near the assay’s limit of detection or a variant in the primer binding region.

**Figure 2:**
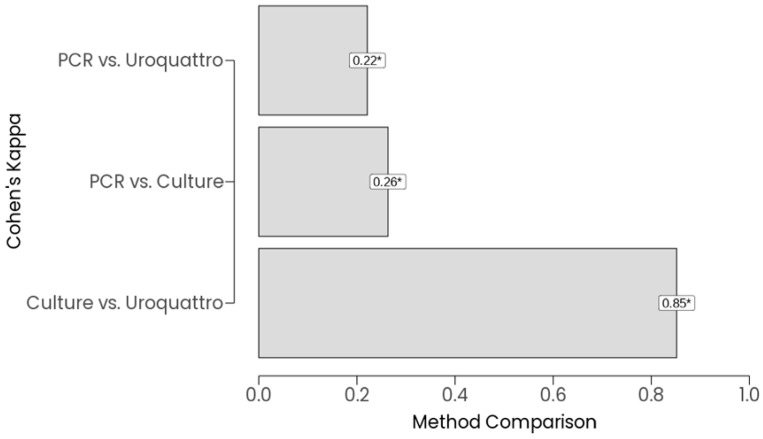
Agreement Between Detection Methods. Bar plot of Cohen’s Kappa values for pairwise agreement between PCR, standard culture, and Uroquattro screening (*N* = 68). Asterisks (*) indicating *p* < 0.05.

Agreement between the three methods was assessed using Cohen’s Kappa on the 68 samples with complete data, as visualized in Figure 2. Pairwise Kappa values indicated varying levels of concordance: PCR vs. standard culture showed fair agreement (Kappa = 0.26, *p* = 0.003), PCR vs. Uroquattro screening also showed fair agreement (Kappa = 0.22, *p* = 0.008), and standard culture vs. Uroquattro screening demonstrated almost perfect agreement (Kappa = 0.85, *p* < 0.01).

### 3.2. Microorganism Prevalence and Agreement

*Escherichia coli* was the most prevalent organism detected by mPCR (37.5%, 95% CI: 27.2–49.0%), followed by *Actinobaculum schaalii* (34.7%, CI: 24.8–46.2%) and *Enterococcus faecalis* (33.3%, CI: 23.5–44.8%). Other frequently detected pathogens included *Aerococcus urinae* (12.5%, CI: 6.72–22.1%), *Klebsiella pneumoniae* (12.5%, CI: 6.72–22.1%), and *Morganella morganii* (11.1%, CI: 5.74–20.4%). Notably, PCR identified fastidious organisms such as *Corynebacterium urealyticum* (8.33%, CI: 3.88–17.0%) and *Streptococcus agalactiae* (5.56%, CI: 2.18–13.4%), which were undetected by culture. In contrast, culture detected *Escherichia coli* at a significantly lower rate (18.1%, CI: 10.9–28.5%) and failed to identify *Actinobaculum schaalii*, *Aerococcus urinae*, and *Corynebacterium urealyticum* entirely. *Klebsiella pneumoniae* (8.33%, CI: 3.88–17.0%) and *Enterococcus faecium* (4.17%, CI: 1.43–11.5%) were among the few pathogens recovered by culture. Non-overlapping confidence intervals for prevalent organisms (e.g., *Actinobaculum schaalii* PCR: 24.8–46.2% vs. culture: 0–5.07%) further confirmed PCR’s higher microorganism detection frequency in this cohort, which reflects differences in biological targets rather than established diagnostic superiority. Discordant results may reflect differences in biological targets (viable growth versus nucleic acid detection), prior antibiotic exposure, fastidious organisms, low bacterial load, or contamination. Rare culture-positive/PCR-negative cases (e.g., Citrobacter gillenii) may reflect organisms not covered by the panel, concentrations near the assay’s limit of detection, or pre-analytical variability.

For microorganisms detected by both PCR and standard culture within the same patient, Spearman’s rank correlation assessed the relationship between cycle threshold (Ct) values and colony counts (count power). Among the matched detections, a weak negative correlation was observed (rho = −0.168, *p* = 0.365), suggesting no statistically significant association.

**Figure 3:**
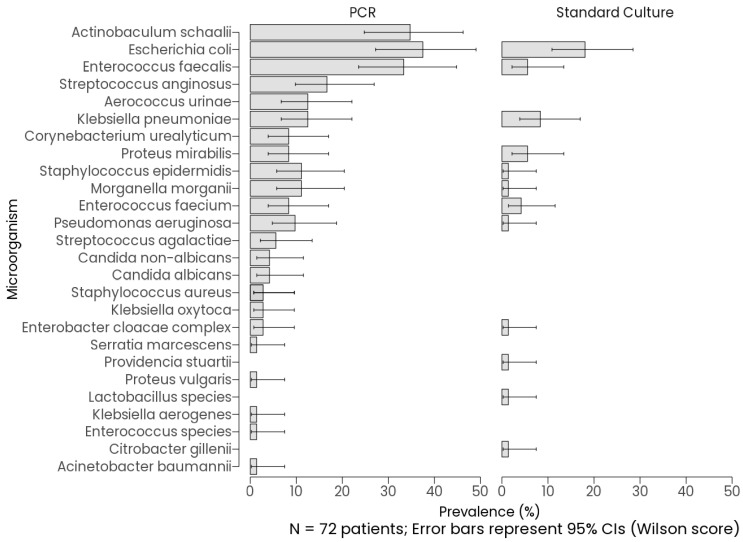
Prevalence of Microorganisms by Method (PCR vs. Standard Culture).

To provide additional quantitative context, median cycle threshold (Ct) values were examined for the most frequently detected organisms (Figure 3). *Escherichia coli* (*n* = 27) showed a median Ct of 21.8 (IQR: 14.9–25.7), *Klebsiella pneumoniae* (*n* = 9) a median Ct of 19.3 (IQR: 16.4–22.5), *Enterococcus faecalis* (*n* = 24) a median Ct of 27.5 (IQR: 21.2–31.6), and *Actinobaculum schaalii* (*n* = 25) a median Ct of 30.4 (IQR: 28.3–31.1). Fastidious organisms such as *Aerococcus urinae* (*n* = 9, median Ct = 33.7, IQR: 30.6–34.2) and *Streptococcus anginosus* (*n* = 12, median Ct = 30.6, IQR: 28.2–31.0) exhibited consistently higher Ct values, suggesting lower bacterial nucleic acid loads compatible with their known growth-limiting characteristics. Among *E. coli* detections stratified by culture concordance, median Ct values were lower in culture-positive samples (median 15.9, IQR: 12.4–21.9, *n* = 12) than in culture-negative samples (median 24.4, IQR: 20.2–27.8, *n* = 15), suggesting that higher molecular loads were more likely to yield concordant culture results. *A. schaalii* was detected exclusively by mPCR across 25 samples with Ct values ranging from 19.2 to 34.0, consistent with its fastidious growth requirements and predictable absence from conventional aerobic culture.

**Figure 4:**
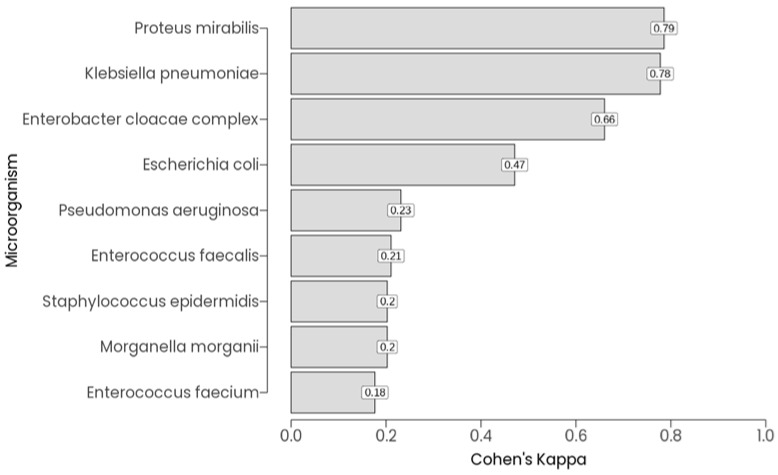
Agreement between PCR and culture was evaluated by microorganism using Cohen’s Kappa, based on binary detection across all 72 patients.

Agreement between PCR and culture was evaluated per microorganism using Cohen’s Kappa, based on binary detection across all 72 patients (Figure 4). Kappa values varied widely by organism. *Klebsiella pneumoniae* exhibited almost perfect agreement (Kappa = 0.778, *p* < 0.01), and *Proteus mirabilis* showed similarly high concordance (Kappa = 0.786, *p* < 0.01). *Escherichia coli* demonstrated moderate agreement (Kappa = 0.471, *p* < 0.01), while *Enterobacter cloacae complex* showed substantial agreement (Kappa = 0.660, *p* < 0.01). In contrast, organisms like *Enterococcus faecalis* (Kappa = 0.211, *p* = 0.0036), *Pseudomonas aeruginosa* (Kappa = 0.231, *p* = 0.0022), *Staphylococcus epidermidis* (Kappa = 0.203, *p* = 0.0044), and *Morganella morganii* (Kappa = 0.203, *p* = 0.0044) exhibited fair agreement, with statistically significant but lower concordance. Several microorganisms, including *Candida albicans*, *Actinobaculum schaalii, Streptococcus agalactiae*, *Streptococcus anginosus*, *Klebsiella oxytoca*, *Candida non-albicans*, and *Staphylococcus aureus*, showed complete discordance.

## 4. Discussion

### 4.1. Comparative Analysis of mPCR and SUC Detection Rates

The results of our study provide valuable insights for discussion. Regarding the high detection rate of the multiplex qPCR assay, which detects pathogens in 83.3% of cases, while standard culture SUC detects pathogens in only 47.2%. This is a strong argument in favour of molecular methods in complicated infections, including fastidious and difficult-to-cultivate microorganisms [23,29,30].

The observed level of agreement (Kappa = 0.26, fair agreement) between PCR and SUC in our results should not be interpreted as a limitation of the PCR method, but rather as a reflection of the differences in the principles of the two methods. It is well known that urine culture requires the presence of viable bacteria that can grow in vitro, while PCR detects bacterial genetic material regardless of the viability of the microorganisms. This explains the presence of PCR-positive/culture-negative cases, especially in patients with previous antibiotic therapy, low bacterial load, or infection caused by difficult-to-cultivate pathogens [31]. It is important to emphasize that the study population was characterized by pronounced clinical symptoms, with 81% of patients having dysuria and 83% having clinical evidence of pyelonephritis.

In our study, mPCR identified a higher percentage of polymicrobial infections compared to SUC. In these cases, the careful interpretation of the mixed flora is necessary because of the possible risk of contamination. On the other hand, the presence of severe clinical symptoms (pyelonephritis) in these patients suggests the true pathogenetic role of the detected microbial associations [30]. This observation is in line with the data of Kline (2016) and Haley (2023), who emphasized that the interaction between different bacterial species in polymicrobial urinary tract infections can lead to a more severe course of the disease and enhance the virulence of the microorganisms involved [29,30].

In the present cohort, polymicrobial detection by mPCR was considerably more frequent than by SUC, and the most commonly co-detected organisms: *E. coli*, *E. faecalis*, and *A. schaalii*, included species that are rarely or never recovered by conventional culture under standard aerobic conditions [24]. Among the 26 culture-positive polymicrobial mPCR samples, partial organism-level concordance was high (92.3%), indicating that in the majority of cases at least one mPCR-detected organism was confirmed by culture, while additional species were identified exclusively by the molecular method. The finding that 87.2% of polymicrobial mPCR detections occurred in patients with acute pyelonephritis lends clinical plausibility to the pathogenetic relevance of these mixed findings, although the absence of an independent clinical reference standard and outcome data precludes definitive attribution of aetiological significance to individual co-detected organisms. It should also be acknowledged that the high frequency of polymicrobial mPCR results raises the possibility of over-detection, particularly for organisms such as *A. schaalii* and *S. anginosus* that may represent colonization or low-level carriage rather than active infection. Future studies incorporating quantitative molecular thresholds and clinical endpoints are needed to establish criteria for distinguishing clinically meaningful polymicrobial infection from incidental co-detection.

Stratification of method discordance revealed that the PCR+/SUC− pattern was the predominant discordance type, observed in 27 of 72 samples, substantially exceeding the reverse pattern (PCR−/SUC+, *n* = 1). The absence of statistically significant differences in age, sex, diagnosis, CRP, or WBC between PCR+/SUC− and concordant-positive samples suggests that the discordance is driven primarily by analytical rather than clinical factors, namely, the capacity of mPCR to detect nucleic acid from non-viable, fastidious, or antibiotic-suppressed organisms that fail to grow under standard aerobic culture conditions. This interpretation is supported by the organism profile within the PCR+/SUC− group, which was dominated by *A. schaalii*, a fastidious species invariably missed by conventional culture, alongside *E. coli* and *E. faecalis*, which in these culture-negative cases likely reflect prior antibiotic suppression of viable growth. The striking disparity in Uroquattro positivity between PCR+/SUC− samples and concordant-positive samples further confirms that rapid nephelometric screening, like SUC, is contingent upon bacterial viability and does not compensate for the analytical gap between culture-based and molecular methods [30,32,33]. The non-significant trend toward higher CRP in the PCR+/SUC− group is noteworthy and may suggest ongoing inflammatory responses in patients whose pathogens are detectable only by molecular methods, although this observation requires confirmation in larger cohorts [34]. Formal multivariable modelling of factors associated with each discordance type was not pursued given the modest sample size and the absence of antibiotic exposure data. Future studies with prospectively recorded covariates should employ logistic regression to identify independent predictors of PCR+/SUC− discordance.

In this context, PCR-positive/culture-negative findings should be interpreted cautiously. As discussed above, such discordance is expected given the fundamentally different biological targets of the two methods and may reflect several mechanisms including prior antibiotic suppression of viable growth, low bacterial load, colonization, or residual non-viable DNA. Without an independent clinical reference standard, the study cannot determine which method better reflects true infection status. It is therefore essential to recognize that a positive mPCR result reflects the presence of bacterial nucleic acid rather than confirmed aetiological involvement, and clinical correlation remains indispensable for appropriate therapeutic decision-making.

### 4.2. Comparative Performance of Uroquattro Screening, Standard Culture, and mPCR in Diagnostic Workflows

The results of the present study show that the Uroquattro screening method showed a positive rate (42.6%) comparable to standard urine culture (47.2%), with the difference between the two methods not being statistically significant (*p* = 0.371). These data confirm the significant role of Uroquattro as an effective tool for rapid screening of negative samples and its use to optimize laboratory testing. However, as with SUC, Uroquattro requires viable bacterial growth and therefore misses the same pathogen categories. Notably, a recent clinical validation study of the HB&L system in urolithiasis patients demonstrated high concordance with standard urine culture for preoperative UTI screening, confirming its role as an effective rapid screening tool where bacterial viability is preserved, while also highlighting its limitations in settings where prior antibiotic exposure may suppress viable growth [35]. From a laboratory workflow perspective, the shorter turnaround time of mPCR may provide earlier organism detection results than culture-based methods. However, without outcome assessment, the present study does not evaluate whether earlier molecular detection translates into improved clinical management. At the same time, it is important to emphasize that PCR panels, including the Novaplex used, provide information about the presence of the pathogen, but cannot replace SUC for determining antibiotic susceptibility, which maintains the culture method as an indispensable part of the diagnostic algorithm. As noted above, the clinical interpretation of mPCR results requires distinction between active infection and detection of non-viable or colonizing organisms. However, in the study population with significant clinical symptoms, the balance between potential over-detection and missed viable growth cannot be determined in this study design, and clinical interpretation remains essential.

The high prevalence of fastidious organisms detected exclusively by mPCR, most notably *Actinobaculum schaalii* and *Aerococcus urinae*, raises considerations for empirical antimicrobial prescribing in the context of complicated UTI. *A. schaalii* is intrinsically resistant to fluoroquinolones and trimethoprim-sulfamethoxazole, both of which are commonly used as empirical first-line agents for UTI in many European settings [32], while remaining susceptible to beta-lactams. If a substantial proportion of culture-negative cUTI cases are attributable to such fastidious organisms, standard empirical regimens selected on the basis of local antibiograms derived exclusively from culture-based surveillance may not provide adequate coverage [9]. Incorporating molecular detection into diagnostic workflows could therefore, in principle, inform more targeted empirical therapy and support antimicrobial stewardship by reducing unnecessary broad-spectrum prescribing or identifying cases in which standard empirical regimens are unlikely to be effective [36]. However, it must be emphasized that these considerations remain speculative within the present study design. The absence of clinical outcome data, antimicrobial susceptibility testing for mPCR-only detections, and information on prior antibiotic exposure precludes any firm conclusions regarding the therapeutic impact of molecular findings. Prospective studies incorporating clinical endpoints and treatment response data are required to determine whether mPCR-guided empirical prescribing improves outcomes in patients with complicated UTI.

### 4.3. Mechanistic Evaluation of Discordant Results and Analytical Limitations

The comparative analysis revealed three notable cases of discordance between molecular and culture methods. In the first case, *Candida glabrata* (10^5^ CFU/mL) was not detected by PCR, which could be explained by the specific scope of the multiplex panel—often targeting *Candida albicans* and grouping *Candida nonalbicans*—or by the inherent difficulties in fungal DNA extraction, given the robust chitin structure of the yeast cell wall. Similarly, the failure to detect *Enterococcus faecium* at high microbial counts (10^5^ CFU/mL) suggests the influence of factors such as the presence of PCR-inhibiting substances in the urine such as urea, heparin or high levels of proteins as well as possible rare genetic mutations in the primer binding sites. In the third case, the negative PCR result for *Pseudomonas aeruginosa* at a lower concentration (10^3^ CFU/mL) is likely a function of the analytical limit of detection (LoD) of the assay. Since many multiplex platforms are calibrated for a clinical threshold of ≥10^4^ CFU/mL to prioritize significant bacteriuria, this is because the molecular result effectively differentiates active infection from low-level colonization or environmental contamination. Based on the discrepancies described above, it can be emphasized that while PCR provides rapid diagnostic information, SUC remains essential for identifying pathogens that fall outside the predefined panel.

An inherent limitation of any targeted multiplex PCR platform is that organisms not included in the panel will be missed regardless of their clinical relevance. The Novaplex™ UTI panels cover 23 targets across three panels (Table A3), encompassing the most prevalent Gram-negative and Gram-positive uropathogens as well as selected fastidious species and *Candida* spp. However, several clinically relevant organisms are not represented, including *Proteus vulgaris*, *Serratia marcescens*, *Stenotrophomonas maltophilia*, *Mycoplasma hominis*, *Ureaplasma urealyticum*, and less common *Candida* species such as *Candida auris*. In the present cohort, the single PCR-negative/culture-positive case involved *Enterococcus faecium*, which is included in the panel, suggesting that this instance of reverse discordance was attributable to factors other than panel coverage—possibly a concentration near the assay’s limit of detection or a variant in the primer binding region. Nonetheless, in clinical practice, a negative mPCR result should not be interpreted as excluding infection, particularly in patients with persistent symptoms and negative culture, where organisms outside the panel may be aetiologically relevant. Clinicians should remain aware of the specific panel composition when interpreting molecular results, and the development of broader panels or complementary metagenomic approaches may address this gap in future diagnostic workflows. Indeed, recent multicentre data have shown that targeted next-generation sequencing platforms can identify polymicrobial infections in over 55% of suspected UTI samples—substantially exceeding both culture and panel-based PCR—while simultaneously detecting antibiotic resistance genes, suggesting that sequencing-based approaches may ultimately supersede fixed-panel assays [24].

While the quantitative correlation between cycle threshold (Ct) values and microbial counts (CFU/mL) is of significant clinical interest, it was not within the primary scope of the current study. Detailed investigation of the relationship between genetic load and bacterial viability is the subject of planned future studies that will allow for more precise validation of PCR as a semiquantitative tool. The median Ct values reported in the present study provide preliminary descriptive data in this regard. The observation that *E. coli* culture-positive samples exhibited substantially lower Ct values than culture-negative samples is consistent with the expectation that higher bacterial loads favour culture recovery, whereas organisms detected at higher Ct values, particularly fastidious species such as *A. schaalii* and *A. urinae*, were invariably missed by SUC, reflecting both lower nucleic acid concentrations and inherent culture limitations for these species [30].

Our findings indicate that mPCR yields a higher microorganism detection rate than culture-based methods in this cohort and may be considered a complementary diagnostic approach alongside culture, which remains essential for antimicrobial susceptibility testing. The higher rates are especially important in emergency cases, complicated UTIs, or suspected urosepsis, where rapid pathogen identification is crucial to initiate timely antimicrobial therapy. However, SUC remains an integral part of the diagnostic algorithm to provide phenotypic testing for antibiotic susceptibility and to allow de-escalation or adjustment of therapy.

Analysis of the clinic-laboratory parameters presented in Table 1 demonstrates a lack of statistically significant differences between the group with detected microorganisms and the group with no detection (all *p*-values ≥ 0.4). From a clinical perspective, this parity in inflammatory marker levels and hematological parameters is of critical significance. Median levels of C-reactive protein (CRP) (8 mg/L vs. 10 mg/L) and white blood cell (WBC) counts remained comparable across both subgroups, indicating that the systemic inflammatory response in these patients does not always correlate directly with the results of instantaneous microbial detection.

The absence of significant variations in parameters such as hemoglobin (HGB) and erythrocyte sedimentation rate (ESR) between the two groups suggests a similar overall health profile and disease severity among all subjects. This observation supports the hypothesis that conventional paraclinical investigations, while useful for assessing general health status, lack sufficient predictive value to confirm or exclude a bacterial ethology in complicated urinary tract infections (cUTIs).

The lack of significant differences in inflammatory markers between detection groups suggests that systemic inflammation does not align closely with instantaneous microbiological detection by any single method. These findings are compatible with several explanations, including pre-analytical variability, prior antibiotic exposure, low bacterial load, or organisms with challenging growth requirements; however, the study did not collect antibiotic exposure timing and did not assess outcomes, so these interpretations remain speculative. The similar inflammatory profile observed in the PCR- and SUC-negative group is likely attributable to the presence of fastidious pathogens or the impact of prior antibiotic therapy. Such therapy may suppress bacterial viability below the detection threshold of standard urine culture while leaving the host’s inflammatory response active. Collectively, these findings strongly argue for the necessity of employing molecular methods such as mPCR, which detect bacterial nucleic acid independently of organism viability and can therefore identify a broader spectrum of organisms, including fastidious and antibiotic-suppressed pathogens even in the presence of normal or non-specific paraclinical indicators. This lack of correlation between systemic inflammation and microbiological results is consistent with the evidence synthesized. This phenomenon has been observed in other studies of complicated infections, specifically, Kline et al. [30], Haley et al. [29] and Grigoryan et al. [31].

### 4.4. Study Limitations

Despite the significant findings, certain limitations of this study should be noted. The sample size of 72 patients is a recognized limitation of this study. No formal a priori power calculation was performed, and the cohort size may be insufficient for organism-specific subgroup analyses, where cell counts for individual pathogens were frequently sparse. Moreover, Cohen’s kappa is known to be sensitive to both sample size and the marginal prevalence of the outcome; in small samples with low-prevalence organisms, kappa point estimates may be unstable and associated confidence intervals wide, potentially limiting the reliability of organism-level agreement classifications. The overall method-level agreement estimates (mPCR vs. SUC, mPCR vs. Uroquattro, SUC vs. Uroquattro) are more robust given the full-sample denominators, yet even these should be interpreted with recognition of the modest precision afforded by 72 observations. Results should accordingly be interpreted as exploratory and hypothesis-generating, and confirmation in larger, multicentre cohorts is warranted. However, the study focused primarily on a specific population with pronounced clinical symptoms of complicated urinary tract infections (UTIs), including both cystitis and pyelonephritis; thus, the findings may differ in cases of asymptomatic bacteriuria or uncomplicated infections.

Beyond the clinical characteristics of the cohort, certain methodological constraints regarding the interpretation of molecular data must be addressed. Antibiotic exposure prior to sampling was not systematically recorded, which represents a critical limitation given that pre-sampling antimicrobial therapy is arguably the single most important driver of PCR-positive/culture-negative discordance, a core finding of this study. Antibiotics may suppress bacterial viability below the detection threshold of culture while leaving nucleic acid targets intact for molecular detection, and the magnitude of this effect is dependent on the timing, duration, and class of antimicrobial agent. Without stratification by antibiotic exposure status, it is not possible to determine what proportion of the observed discordance reflects true analytical differences between methods versus the pharmacological suppression of culture yield. Future studies should prospectively record the timing and duration of antibiotic exposure relative to sampling as a key stratification variable. Specifically, future studies should consider logistic regression modelling of method discordance (PCR-positive/culture-negative status as the dependent variable) with antibiotic exposure characteristics, including class, dose, duration, and interval between last dose and urine sampling, as covariates. Such an analysis would permit quantification of the independent contribution of antimicrobial suppression to the observed discordance between molecular and culture-based methods, and would clarify the extent to which mPCR-exclusive detections in this cohort reflect true analytical superiority versus pharmacologically mediated culture failure. Furthermore, while the multiplex qPCR provided rapid qualitative results, a quantitative analysis, specifically the calibration of cycle threshold (Ct) values to exact DNA copy numbers, was not conducted in this initial phase. Our research group is currently addressing this area, and a detailed quantitative validation aimed at differentiating between active infection and microbial colonization is the subject of a forthcoming study. Finally, comparing the molecular assay against SUC, a ‘gold standard’ with known limitations in detecting fastidious or antibiotic-suppressed organisms, may influence the calculated diagnostic accuracy parameters.

A primary consideration in interpreting these results is that the study did not include an independent clinical reference standard or adjudication panel to determine true infection status. Consequently, diagnostic accuracy measures such as sensitivity, specificity, and predictive values could not be calculated. The results therefore reflect differences in microorganism detection and inter-method agreement rather than confirmation that one method is diagnostically superior, and the mechanistic interpretation of discordant findings remains subject to the considerations outlined in Section 4.1.

Given the fundamental difference in biological targets between culture-based and molecular methods (discussed in Section 4.1), discordant results are expected. Without clinical outcome data, the clinical relevance of additional organisms detected exclusively by mPCR cannot be determined, and the possibility of over-detection leading to unnecessary treatment should be considered.

Regarding the study environment and population, the cohort consisted of hospitalized patients with clinical suspicion of complicated UTI in a single centre. This limits generalizability to primary care settings, asymptomatic bacteriuria, screening populations, and catheter-associated infections. The exclusion of certain patient groups and the relatively modest sample size also restrict organism-specific analyses and subgroup inference. It should also be acknowledged that the classification of polymicrobial cultures as clinically relevant versus probable contamination, while now defined by explicit operational criteria, retains an element of clinical judgement. The absence of a formal adjudication panel or blinded independent review of polymicrobial cases introduces the possibility of differential classification. However, the number of samples affected by this criterion was small, and sensitivity of the primary findings to the inclusion or exclusion of borderline polymicrobial cases is expected to be limited.

In addition to these factors, potential pre-analytical variables may have influenced results. Urine sampling technique, transport conditions, and storage duration can affect both culture yield and molecular detection. In addition, the multiplex PCR panel targets a predefined set of microorganisms; therefore, pathogens not included in the panel may be missed, while detected organisms may not necessarily be etiologically relevant.

Another important limitation is that antimicrobial susceptibility testing was only available for culture-positive isolates. As a result, the study could not evaluate whether PCR-guided management would alter therapy, clinical outcomes, or antimicrobial stewardship practices.

Finally, the cross-sectional design did not assess patient outcomes such as treatment modification, clinical resolution, recurrence, or complications. The study therefore evaluates the analytical detection rate of mPCR relative to culture-based methods, but cannot address the clinical utility of the additional molecular detections—that is, whether the organisms identified exclusively by mPCR were aetiologically responsible for the presenting illness and whether their detection would have altered therapeutic decisions or improved patient outcomes. This distinction between analytical performance and clinical utility is fundamental, and prospective studies incorporating treatment response, infection resolution, and antimicrobial stewardship endpoints are essential to determine whether routine incorporation of mPCR into diagnostic workflows confers measurable clinical benefit in the management of complicated UTI.

## 5. Conclusions

The present study demonstrates significant differences in microorganism detection frequency and inter-method agreement between mPCR, standard culture, and nephelometric screening. PCR allows the identification of uropathogens in a significant proportion of cases that remain culture-negative with classical microbiological methods, despite the presence of clinical symptoms, reflecting the fundamental difference in biological targets between the methods rather than an inherent inferiority of culture-based approaches. While multiplex PCR provides rapid identification, it has inherent limitations in providing comprehensive information on antibiotic susceptibility compared to phenotypic testing. However, the low degree of agreement between the methods, as well as the limitations of PCR to provide information on the antibiotic susceptibility of the isolated pathogen, necessitate cautious interpretation of the results and emphasize the need for a combined diagnostic approach.

While this study demonstrates a higher detection frequency by mPCR in this cohort, future research is needed to refine the quantitative correlation between genetic load (Ct values) and microbial viability. Future outcome-based studies are required to determine whether incorporating mPCR into routine workflows improves clinical decision-making and antimicrobial stewardship.

## Figures and Tables

**Table 1:** Descriptive Statistics of Study Participants. Continuous variables are presented as median (IQR); categorical variables as *n* (%). *p*-values are from Wilcoxon rank-sum tests (continuous) or Fisher’s exact tests (categorical).

Characteristic	Overall (*N* = 72)	No Detection (*N* = 11)	Detected (*N* = 61)	*p*-Value
Age (years)	58 (40, 71)	58 (25, 71)	58 (42, 70)	>0.9
Female	53 (74%)	9 (82%)	44 (72%)	0.7
Diagnosis				0.4
Acute Pyelonephritis	60 (83%)	8 (73%)	52 (85%)	
Acute Cystitis	12 (17%)	3 (27%)	9 (15%)	
*Symptoms*				
Cloudiness	49 (68%)	6 (55%)	43 (70%)	0.3
Febrile	24 (33%)	4 (36%)	20 (33%)	>0.9
Vomiting	10 (14%)	1 (9.1%)	9 (15%)	>0.9
Dysuria	58 (81%)	9 (82%)	49 (80%)	>0.9
Pollakiuria	49 (68%)	6 (55%)	43 (70%)	0.3
Urgency	22 (31%)	5 (45%)	17 (28%)	0.3
Enuresis	1 (1.4%)	0 (0%)	1 (1.6%)	>0.9
Haematuria	4 (5.6%)	1 (9.1%)	3 (4.9%)	0.5
Suprapubic Pain	29 (40%)	5 (45%)	24 (39%)	0.7
Succussion	20 (28%)	3 (27%)	17 (28%)	>0.9
Smell	20 (28%)	2 (18%)	18 (30%)	0.7
Urine Colour Change	59 (82%)	8 (73%)	51 (84%)	0.4
Congenital Abnormalities	10 (14%)	0 (0%)	10 (16%)	0.3
Obstruction	6 (8.3%)	2 (18%)	4 (6.6%)	0.2
*Laboratory Values*				
WBC (×10^9^/L)	8.7 (6.5, 10.8)	9.5 (7.8, 11.1)	8.6 (6.5, 10.8)	0.5
RBC (×10^12^/L)	4.41 (3.95, 4.83)	4.40 (3.93, 4.77)	4.41 (3.97, 4.87)	>0.9
HGB (g/L)	125 (112, 137)	128 (111, 140)	124 (112, 135)	0.5
ESR (mm/h)	26 (14, 40)	22 (7, 37)	27 (15, 40)	0.5
CRP (mg/L)	8 (5, 31)	10 (6, 87)	8 (4, 31)	0.4
Urea (mmol/L)	6.3 (4.3, 8.6)	6.5 (4.1, 9.7)	6.1 (4.3, 8.2)	0.8
Urine Specific Gravity	1.015 (1.010, 1.025)	1.015 (1.010, 1.020)	1.015 (1.010, 1.025)	0.4
Urine pH	5.50 (5.00, 6.00)	5.50 (5.00, 6.00)	5.50 (5.00, 6.00)	0.8

Statistical significance was determined using the Mann–Whitney U test for continuous variables and the Chi-square or Fisher’s exact test for categorical variables. Continuous variables are expressed as median (interquartile range, IQR). Categorical variables are expressed as count (percentage). WBC: White Blood Cells; RBC: Red Blood Cells; HGB: Haemoglobin; ESR: Erythrocyte Sedimentation Rate; CRP: C-reactive protein.

## Data Availability

The original contributions presented in this study are included in the article. Further inquiries can be directed to the corresponding author.

## Abbreviations

The following abbreviations are used in this manuscript:

cUTI	Complicated Urinary Tract Infection
mPCR	Multiplex Real-Time PCR
SUC	Standard Urine Culture
EAU	European Association of Urology
IDSA	Infectious Diseases Society of America
MALDI-TOF	Matrix-Assisted Laser Desorption/Ionization Time-of-Flight
LoD	Limit of Detection
CFU/mL	Colony Forming Units per millilitre
EQUC	Extended Urine Culture protocols
NGS	Next-Generation Sequencing
Ct	Cycle threshold
WBC	White Blood Cells
RBC	Red Blood Cells
HGB	Hemoglobin
ESR	Erythrocyte Sedimentation Rate
CRP	C-reactive protein
IQR	Interquartile range
RIMU	Research Institute of Medical University of Plovdiv
IIST	Institute for Innovation and Smart Technology
EMB	Eosin-Methylene Blue
MuDT™	Multiple Detection Temperatures
CI	Confidence Interval
κ (Kappa)	Cohen’s kappa
df	Degrees of freedom
ρ (Spearman’s ρ)	Spearman’s rank correlation coefficient
HIV	Human Immunodeficiency Virus
Cat. No.	Catalog Number
BA	Blood Agar
SD	Standard Deviation
r	Correlation coefficient
N/n	Total population size/Subsample size
μL	Microliter

## Appendix A

**Table A1:** Cross-tabulation of mPCR detection classification (monomicrobial, polymicrobial, negative) against standard urine culture (SUC) result (*N* = 72).

mPCR Classification	Culture-Negative	Monomicrobial Culture	Polymicrobial Culture	Total
Negative	11	1	0	12
Monomicrobial	6	7	0	13
Polymicrobial	21	22	4	47
Total	38	30	4	72

**Table A2:** Cross-tabulation of mPCR and standard urine culture (SUC) binary detection results (*N* = 72).

	SUC Positive	SUC Negative	Total
mPCR positive	33 (45.8%)	27 (37.5%)	60 (83.3%)
mPCR negative	1 (1.4%)	11 (15.3%)	12 (16.7%)
Total	34 (47.2%)	38 (52.8%)	72 (100%)

**Table A3:** Microorganisms targeted by the Novaplex™ UTI Panel Assays (Seegene).

Panel	Target Organism	Gram Classification
Panel 1	*Escherichia coli*	Gram-negative
Panel 1	*Klebsiella pneumoniae*	Gram-negative
Panel 1	*Proteus mirabilis*	Gram-negative
Panel 1	*Pseudomonas aeruginosa*	Gram-negative
Panel 1	*Enterobacter* spp.	Gram-negative
Panel 2	*Enterococcus faecalis*	Gram-positive
Panel 2	*Enterococcus faecium*	Gram-positive
Panel 2	*Staphylococcus aureus*	Gram-positive
Panel 2	*Staphylococcus saprophyticus*	Gram-positive
Panel 2	*Staphylococcus epidermidis*	Gram-positive
Panel 2	*Streptococcus agalactiae* (GBS)	Gram-positive
Panel 2	*Actinotignum (Actinobaculum) schaalii*	Gram-positive
Panel 2	*Candida albicans*	Fungal
Panel 2	*Candida* spp. (non-albicans)	Fungal
Panel 3	*Acinetobacter baumannii*	Gram-negative
Panel 3	*Aerococcus urinae*	Gram-positive
Panel 3	*Citrobacter freundii*	Gram-negative
Panel 3	*Citrobacter koseri*	Gram-negative
Panel 3	*Corynebacterium urealyticum*	Gram-positive
Panel 3	*Morganella morganii*	Gram-negative
Panel 3	*Pantoea agglomerans*	Gram-negative
Panel 3	*Providencia stuartii*	Gram-negative
Panel 3	*Streptococcus anginosus*	Gram-positive
